# Identification of *TMEM217* as a novel prognostic biomarker and potential therapeutic target in acute myeloid leukemia

**DOI:** 10.1016/j.gendis.2023.06.010

**Published:** 2023-07-16

**Authors:** Yunying Yao, Zhizhou Xia, Min Wu, Bo Jiao, Jiaming Gao, Donghe Li, Xi Xie, Pengfei Xu, Jiaoyang Li, Lei Yan, Ruibao Ren, Ping Liu

**Affiliations:** a Shanghai Institute of Hematology, State Key Laboratory for Medical Genomics, National Research Center for Translational Medicine, Collaborative Innovation Center of Hematology, Ruijin Hospital affiliated with Shanghai Jiao Tong University School of Medicine, Shanghai 200025, China; b International Center for Aging and Cancer, Hainan Medical University, Haikou, Hainan 570102, China

Despite remarkable advances in molecular and cell biology of acute myeloid leukemia (AML), AML patients still frequently relapse and have low 5-year overall survival (OS) rates.^1^ It is worth noting that a recent study from the registry or clinical trial compilation has reported an improvement in the OS of adult AML patients, especially those under 60 years of age.^2^ There is an urgent need to unveil more accurate and sensitive biomarkers to improve the survival of AML patients. Gene expression profiling played a pivotal role in hematology and provided crucial insights into the biology of AML.^3^ Here, we identified *TMEM217* as a promising biomarker for prognostic prediction especially for AML patients younger than 60 years of age, and as a target for developing innovative treatments for AML.

Based on the TCGA-LAML cohort, we first defined two clusters (“Cluster 1” and “Cluster 2”) using consensus clustering analysis to select features and extract candidate AML prognosis-related genes (PRGs) (Fig. S1A, B). The PCA showed that samples in two clusters could be visually distinguished (Fig. S1C), and some of the important clinical characteristics including patients' age, FAB classification, and NPM1 mutants were significantly different between the two clusters (Fig. 1A and Table S1). A Kaplan–Meier survival analysis indicated that patients in Cluster 1 exhibited a significantly longer OS compared with Cluster 2 (Fig. 1B), and a total of 913 DEGs from 19,905 genes were identified between the two clusters (Fig. 1C). Subsequently, 464 candidate PRGs were identified by subgroup analysis method and trained by Lasso-penalized Cox regression model (Fig. S1D, E), which winnowed the list down to seven genes: *AEBP1*, *C20orf203*, *ENPP7P10*, *LPO*, *PTPRU*, *TMEM217*, and *UCA1*. The expression of those seven genes was significantly different between AML patients from the TCGA-LAML cohort and normal controls from the GTEx cohort (Fig. S1F). Univariate Cox regression analysis of seven genes revealed that high expression of *C20orf203*, *PTPRU*, and *TMEM217* was associated with a significantly worse OS, while high expression of *AEBP1*, *ENPP7P10*, *LPO*, and *UCA1* was associated with a significantly better OS (Fig. 1D). Multivariate Cox regression analysis of seven genes further indicated that *TMEM217* and *UCA1* were two independent risk factors for OS relative to the other five genes (Fig. 1D). A Kaplan–Meier analysis also indicated that high expression of *TMEM217* was associated with poor prognosis in the TCGA-LAML cohort (Fig. 1E), which was validated in GSE12417-AML, TARGET-AML, and Beat-AML cohorts (Fig. S1G–I), respectively. By contrast, high *UCA1* expression was associated with favorable outcomes (Fig. S1J–L). Given that *UCA1* has been well-studied as an oncogene that contributes to the proliferation capacity of AML cells,^4^ we focused our study on *TMEM217*.

**Figure 1:**
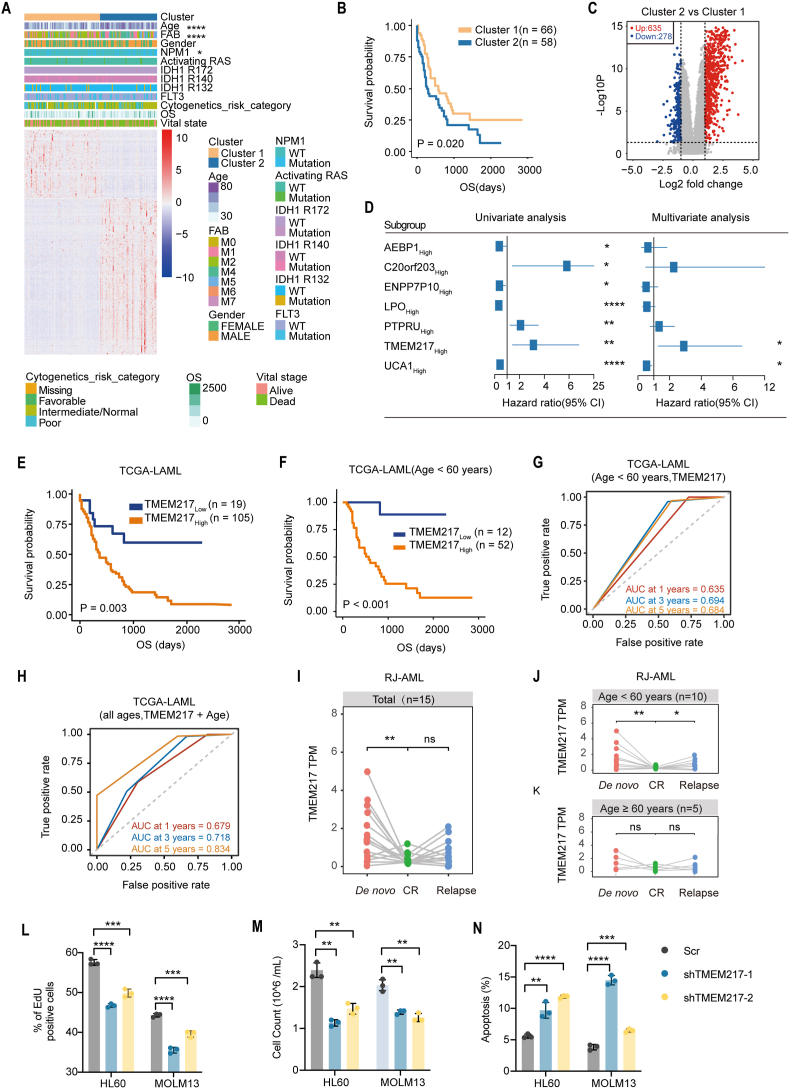
Identification of *TMEM217* as a prognosis biomarker and potential therapeutic target of acute myeloid leukemia (AML). **(A)** Heatmap of association between expression levels of two clusters and their clinical characteristics. **(B)** Kaplan–Meier curve analysis showed significantly different OS between Cluster 1 and Cluster 2 (*P* = 0.020). **(C)** The 913 differentially expressed genes (DEGs) were displayed between Cluster 1 and Cluster 2 by volcano plot. The red (*n* = 635) and blue (*n* = 278) plots represent the genes up-regulated and down-regulated in Cluster 2 relative to Cluster 1, respectively. **(D)** Forest plot of seven candidate genes in subgroups analysis with univariate and multivariate Cox analysis to estimate clinical prognostic value between low and high expression subgroups of each gene. The length of the horizontal line represents the 95% confidence interval for each subgroup. The box represents the hazard ratio (HR) of all patients. The vertical solid line represents HR = 1.0. HR < 1.0 indicates that low expression is a favorable prognostic biomarker. **(E)** Kaplan–Meier curves for patients with high and low *TMEM217* expression in the TCGA-AML cohort. Patients in the high *TMEM217* expression subgroup have worse OS than those in the low subgroup. The log-rank tests produced overall *P* values. n, number. **(F)** Patients younger than 60 years of age with high expression of *TMEM2*17 had worse prognostic survival than those in the low expression group (*P* < 0.001). **(G)** Time-dependent ROC curve analysis based on the expression of *TMEM217* in the patients younger than 60 years group. **(H)** Time-dependent ROC curve analysis was obtained using the prognostic model constructed based on *TMEM217* expression and age in the TCGA-AML all-ages group. **(I–K)** Pairwise comparisons of *TMEM217* expression in fifteen RJ-AML patients (I), ten RJ-AML patients younger than 60 years of age (J), and five patients older than 60 years of age (K) who achieved CR and relapse from *de novo.***(L)** Cell proliferation of each cell line after *TMEM217* knockdown was measured by EdU assay 1 h after plating. **(M)** Cell proliferation of each cell line using the cell counting method. **(N)** Flow cytometry examined the apoptosis of each cell line using Annexin V/PI staining.

Known risk factors for poor prognosis in AML include age (older than 60 years of age) and genetic aberrations,^5^ four multivariate Cox regression models were constructed to evaluate the prognostic independence of *TMEM217* in the TCGA-LAML cohort. After adjusting for age, *FLT3*, *IDH1*, activating *RAS*, and *NPM1* mutations, we still detected significant differences in OS of AML patients between the low and high *TMEM217* expression subgroups (Fig. S2A). We then assessed the influence of TMEM217 expression on the prognosis of AML in both young and old patient groups (young/old groups: younger/older than 60 years of age). The results indicated that high *TMEM217* expression and IDH1_R132^mut^ were significant in the young group (Fig. S2B), while high *TMEM217* expression was not significant in the old group (Fig. S2C). When an interaction term for high *TMEM217* expression and IDH1_R132^mut^ was included in the young group, there were no significant interaction effects (Fig. S2D). Similarly, our analysis based on the TCGA-LAML cohort showed that only in the young group, patients with high *TMEM217* expression had a significantly lower probability of survival than low *TMEM217* expression (Fig. 1F; Fig. S2E, F). These findings were validated in GSE12417-AML (Fig. S2G, H) and Beat-AML cohorts (Fig. S2I, J). Taken together, these findings support that high *TMEM217* expression is an independent prognosis biomarker for AML patients younger than 60 years of age.

To assess the prognosis prediction accuracy of *TMEM217* expression for OS, we first constructed three time-dependent *TMEM217* ROC models for the TCGA-LAML cohort. The results showed that *TMEM217* expression had prognostic prediction accuracy in AML patients, especially for the young group (Fig. 1G; Fig. S3A, B). Moreover, a modified prognosis model developed by the combination of age and *TMEM217* expression showed higher accuracy than *TMEM217* expression alone (Fig. 1H; Fig. S3A). These results were also validated in three AML cohorts (Figs. S3C–K). The TARGET-AML cohort data did not include any patients over 60 years of age, therefore only the age <60 figure was presented.

We next conducted a longitudinal transcriptomics analysis of a total of 23 AML patients at our center (RJ-AML) that presented with *de novo*, complete remission (CR), and relapse three stages (15 patients collected fully paired samples at the three stages — 15-patients group, 18 patients collected paired samples at the *de novo* and CR stages — 18-patients group, and 21 patients collected paired samples at the CR and relapse stages — 21-patients group). The heatmap of transcriptomics and clinical characteristics for 23 patients are presented in Figure S4A. The expression level of *TMEM217* of those RJ-AML patients had no significant difference even though they harbor different genetic mutations, in different age groups, or different gender (Fig. S4B–D).

To explore potential correlations between *TMEM217* expression and AML stages, we first compared the expression pattern of *TMEM217* in the 15-, 18-, and 21-patients groups, respectively. The expression level of *TMEM217* at the CR stage was significantly lower compared with the *de novo* stage. Notably, the *TMEM217* expression level later increased at the relapse stage (Fig. 1I; Fig. S4E, H).

Given our findings about age and *TMEM217* expression for predicting OS, we subsequently also categorized the 15-, 18-, and 21-patients groups into young and old subgroups to investigate the potential correlation between *TMEM217* expression and disease stages specifically in the young group. Significant differences in *TMEM217* expression between CR and relapse in the young subgroups were detected (Fig. 1J; Fig. S4F, I), while no difference was found in the old subgroups (Fig. 1K; Fig. S4G, J). These results indicated that *TMEM217* expression was associated with AML clinical stages for patients younger than 60 years of age.

To explore the potential biological functions of *TMEM217* in AML, two shRNAs targeting *TMEM217* were designed and stably transfected two AML cell lines: HL60 (derived from AML-M2) and MOLM13 (derived from AML-M5) (Fig. S5A, B). *TMEM217* knockdown in both two cell lines led to significant inhibition of proliferation (Fig. 1L, M) and promotion of apoptosis (Fig. 1N).

Taken together, these results suggested that the biological processes affected by *TMEM217* are related to AML cell apoptosis and proliferation, but the function and molecular mechanisms of *TMEM217* affecting hematopoietic malignancies need to be further elucidated.

## Ethics declaration

The publicly available data in this study were exempt from both ethics approval and informed consent. Users can download relevant data for free for research and publish relevant articles. The collection of the specimens in the RJ-AML cohort was approved by the Ethics Committee from Ruijin Hospital, Shanghai, China, and written informed consents for specimen collection and research were obtained.

## Author contributions

P.L. and R.R. designed and supervised the study. Y.Y. analyzed and managed the data. P.L. and Z.X. supervised data collection. Z.X., P.L., and B.J. helped with biological experiments. Y.Y. and Z.X. prepared the first draft of the manuscript. R.R., P.L., Y.Y., and Z.X. reviewed and edited the paper. M.W., J.G., D.L., X.X., P.X., J.L., and L.Y. discussed the paper together. P.L. and R.R. read and approved the final manuscript.

## Conflict of interests

Yunying Yao, Zhizhou Xia, Min Wu, Bo Jiao, Jiaming Gao, Donghe Li, Xi Xie, Pengfei Xu, Jiaoyang Li, Lei Yan, Ruibao Ren, Ping Liu declare that they have no conflict of interests.

## Funding

This work was supported by the Key Project of the 10.13039/501100001809National Natural Science Foundation of China (No. 82230088 to R.R.), 10.13039/501100001809National Natural Science Foundation of China (No. 81870112, 82170147 to R.R.; No. 81970134, 82170111 to P.L.; No. 82200200 to Z.X.), Shanghai Science and 10.13039/100006180Technology Development Funds (China) (No. 20Z11900200 to R.R.; No. 18ZR1423600 to P. L), Shanghai Collaborative Innovation Program on Regenerative Medicine and Stem Cell Research (China) (No. 2019CXJQ01 to R.R.), the Samuel Waxman Cancer Research Foundation (to R.R.), the Innovative Research Team of High-level Local Universities in Shanghai, China (to R.R.), and the National Key Research and Development Program of China (No. 2022YFC2705004 to P. L.).

## References

[1] Stratmann S., Yones S.A., Mayrhofer M. (2021). Genomic characterization of relapsed acute myeloid leukemia reveals novel putative therapeutic targets. Blood Adv.

[2] Ellison L.F. (2016). Increasing survival from leukemia among adolescents and adults in Canada: a closer look. Health Rep.

[3] Lilljebjörn H., Orsmark-Pietras C., Mitelman F., Hagström-Andersson A., Fioretos T. (2022). Transcriptomics paving the way for improved diagnostics and precision medicine of acute leukemia. Semin Cancer Biol.

[4] Hughes J.M., Legnini I., Salvatori B. (2015). C/EBPα-p30 protein induces expression of the oncogenic long non-coding RNA UCA1 in acute myeloid leukemia. Oncotarget.

[5] Döhner H., Wei A.H., Appelbaum F.R. (2022). Diagnosis and management of AML in adults: 2022 recommendations from an international expert panel on behalf of the ELN. Blood.

