# Supplementary material for: Identification of TMEM217 as a novel prognostic biomarker and potential therapeutic target in acute myeloid leukemia
Source: Genes Dis. 2023 Jul 16;11(4):101037. doi: 10.1016/j.gendis.2023.06.010 (PMC10950817; doi:10.1016/j.gendis.2023.06.010)
Supplement: Multimedia component 1 [file mmc1.docx]

**Supplementary materials**

**Identification of *TMEM217* as a novel prognostic biomarker and potential therapeutic target in acute myeloid leukemia**

Yunying Yao^*^, Zhizhou Xia^*^, Min Wu, Bo Jiao, Jiaming Gao, Donghe Li, Xi Xie, Pengfei Xu, Jiaoyang Li, Lei Yan, Ruibao Ren^#^, Ping Liu^#^

**Supplementary Data**

**Materials and Methods**

**Samples collection and data selection**

In the RJ-AML cohort, 59 bone marrow samples of 23 AML patients at *de novo*, complete remission, and relapse stages are consecutively enrolled from the Shanghai Institute of Hematology between March 2015 and March 2020, according to the NCCN Guidelines [1]. The median age of all patients was 47 years (ranging from 26-75 years). The patients were scheduled for cytarabine combined with anthracycline standard induction and a cytarabine-based consolidation chemotherapy. The median first CR maintenance follow-up time was 75.5 days, and the median time to relapse was 315 days.

135 AML cases in the TCGA-LAML cohort, 187 AML cases in the TARGET-AML cohort, and 70 healthy human samples (normal control) in the GTEx cohort were all retrieved via the University of California Santa Cruz (UCSC) Xena database [2]. The GSE12417 cohort (GPL97), contains 162 cases of AML obtained from Gene Expression Omnibus (GEO) [3]. The further information about the 200 AML cases in the Beat-AML cohort was concluded in the data availability section of Tyner et al [4].

The AML patients included in the analysis must meet all three of the following criteria: (1) Non-M3 AML samples; (2) contained relevant clinical information such as age, survival time, and survival status; and (3) had no missing values in gene expression data in any samples.

**Unsupervised Consensus Cluster Analysis**

The ConsensusClusterPlus R package [5] was used to perform K-means-based consensus clustering to identify distinct clusters (subtypes) of 135 AML samples based on TCGA-LAML transcriptomic profiles. Clustering was performed using the pam algorithm, with 80% item resampling and 1000 repetitions; a distance matrix of the transcriptomic profiles was calculated by Spearman correlation analysis. The cluster-consensus plot and circular manhattan (CM) plot at each k were obtained. The high average pairwise consensus value of cluster-consensus plot indicated high stability of clustering, which was used to select representative samples for further analysis. The principal component analysis (PCA) and hierarchical clustering analysis (HCA) were used to analyze the sample between the clusters. Heatmaps of hierarchical clustering analysis were plotted using pheatmap (RRID:SCR_016418) R package in this study.

**Survival Analysis**

The overall survival (OS) was analyzed by Kaplan-Meier (KM) survival based on a log-rank test using the survival (https://cran.r-project.org/package=survival) and survminer (https://cran.r-project.org/package=survminer) R packages.

**Differential Expression Analysis**

The Wilcoxon test was applied to identify differences between the clusters. All statistical tests were two-sided, with P < 0.05 indicating significance. The cutoff value for screening differentially expressed genes (DEGs) was |log2 (fold change) | ≥ 1 and P < 0.05. False discovery rate (FDR) correction [6] was performed on the P values. The volcano plot of DEGs was plotted using ggplot2 R package.

**Subgroup analysis**

The R package maxstat (<https://CRAN.R-project.org/package=maxstat>) was applied iteratively to tests all possible cut points to find the one achieving the maximum log-rank test statistic of OS, was used to dichotomize expression level, and 135 AML patients were then grouped into low and high expression subgroups in each DEG. The genes included in DEGs and were also significant in log-rank tests (P < 0.05) of OS between low and high expression subgroups were designated as AML prognosis-related genes (PRGs).

**Identification of prognosis biomarker for AML**

PRGs were selected to identify the prognosis biomarker of AML by constructing the prognostic risk model. The most stable subset of PRGs was selected by Lasso-penalized Cox regression analysis using the glmnet R package [7] with a penalized maximum likelihood estimator performed by 10 bootstrap replicates, and the optimally weighted coefficients were identified by the regularization parameter lambda via the 1-SE criteria. Univariate and multivariate Cox regression analyses were conducted to evaluate the prognostic value of PRGs that affect AML patient OS. Hazard Ratios (HR) of univariate and multivariate Cox regression analysis were obtained through comparisons between high and low expression subgroups for each PRG. The P < 0.05 of PRGs in multivariate Cox regression analysis were designated as AML prognosis biomarkers for further analysis.

**Association between prognosis biomarkers and clinical characteristics of AML**

Multivariate Cox regression analysis was conducted to evaluate the independence of prognosis biomarkers and clinical characteristics for AML. Interaction analyses between prognosis biomarkers and clinical characteristics were evaluated by introducing interaction terms in multivariable Cox regression analysis. The Wilcoxon test was applied to explore the prognosis biomarkers expression differences in different clinical characteristics groups (such as age, gender, clinical stages, etc.).

**Construction of prognosis prediction models of prognosis biomarkers**

Time-dependent receiver operating characteristic (ROC) curves were used to evaluate the prognosis prediction accuracy of prognosis biomarker using the survival ROC R package [8], and the true positive rate (sensitivity), false positive rate(1-specificity), and area under the curve (AUC) values were determined.

**RNA-sequencing (RNA-seq) analysis**

For the RJ-AML cohort, RNA was extracted from bone marrow using the AllPrep^®^ DNA/RNA Mini kit (50). Sample purity was detected by Nanodrop8000 from Thermo Company. Sample concentration was detected by Qubit3 of Invitrogen Company. Sample integrity and library fragment size were measured by Agilent's 4200 tape station. RNA libraries were constructed using KAPA mRNA-HyperPrep Kit (KAPA Biosystems, American). Paired-end 2 x 150 bp RNA sequencing was performed on Illumina NovaSeq 6000 (Illumina, USA) at National Research Center for Translational Medicine (Shanghai). Raw sequencing reads were quality controled by FASTQC (bioinformatics.Babraham.ac.UK/projects/fastqc/, v0.11.9) and quality-trimmed by trimmomatic [9]. RNA sequence data were aligned to the hg38 human reference genome using STAR (Spiced Transcripts Alignment to a Reference) [10], and uniquely mapped reads were extracted for gene expression quantified by HTSeq software [11].

**Cell culture**

The AML cell lines HL-60 and MOLM13 were bought from ATCC and cultured in RPMI 1640 (Basal Media, Shanghai, China) with 10% (v/v) FBS (ThermoFisher, Waltham, MA, USA) in a humidified incubator at 37 °C under 5% CO2. All cell lines were authenticated via STR profiling.

**EdU incorporation and detection**

Cell proliferation were assessed by EdU Assay (ThermoFisher, MA, USA, Catalog # C10419) performed according to the manufacturer's introductions. Briefly, cells were seeded into 6-well cell plates (5×10^5 cells/well) and the Click-iT EdU diluted in DMSO was added at a final concentration of 10 mM for 1h of incubation. The cells, recovered by pipetting and aspiration, were fixed in 100 μL of Click-it fixative for 15 min at room temperature and washed twice with 1 ml of D-PBS containing 1% FBS. Then the cells were resuspended in 100 μl of permeabilization buffer for 15 min at room temperature. The components of the Click reaction were as follows (for 1 reaction): D-PBS 438 μL, Copper (II) 10 μL, Alexa Fluor™ 647 Azide 2.5 μL, Reaction Buffer Additive 50 μL. The reagents were added in the same order as mentioned above, and the solution was mixed between additions. Subsequently, the cells were resuspended in 500 μL of the mixed solution and incubated for 30 min in the dark at room temperature. Finally, the cells were washed once before analyzing on a flow cytometer.

**Cell counting**

We used cell counting method to directly determine the proliferation ability of the cells. 5×10^5 cells were seeded in each well of a 12-well plate and cultured for 48 hours. The cells were then counted using Countstar automatic cell counter (Alit Biotech, Shanghai). The experiment was repeated at least twice and similar trends were observed in each repetition of the experiment.

**Plasmid construction**

The shRNAs against human *TMEM217* were obtained from GenePharma Corporation (Shanghai, China) and the sequences used for *TMEM217* RNAi experiments are as follows:

Scrambled sequence:

TTCTCCGAACGTGTCACGTTTCTCGAGAAACGTGACACGTTCGGAGAA

shTMEM217-1: CGTAATACAAATCCTCACCAA

shTMEM217-2: GCATCTTCTCTGTCCTTAATA

Both shRNAs were cloned into pLKO.1 (Addgene 10878). All constructs were verified by Sanger sequencing via Sunnybio (Shanghai, China).

**Lentivirus production and generation of stable cell lines**

For lentivirus production, lentiviruses were generated following the protocol from Addgene [13]. Briefly, a 6 cm dish with 80% confluent HEK293T cells was transfected using 1 μg pLKO.1 shRNA plasmid, 750 ng psPAX2 packaging plasmid, 250 ng pMD2.G envelope plasmid, 500 µL serum-free Opti-MEM^®^ Medium and 10 μL Lipofectamine^®^ 2000 (Thermo) transfection reagent. After 48 hours’ transfection, viral supernatants were filtered through a 0.22 μm low protein binding membrane (Millipore, Darmstadt, Germany). Then, targeted cells were transduced in 6-well plates and treated with 2 μg/mL puromycin for a 2-week selection. *TMEM217* Knockdown efficiency was determined by Western blot following the protocol described before [12]. The anti-TMEM217 was bought from ThermoFiser (Catalog # PA5-112756).

**Cell Apoptosis assay**

Cell apoptosis was examined using Annexin V-APC Apoptosis Detection Kit (eBioscience) and following the manufacturer’s guidelines. FACS data were analyzed via FlowJo (TreeStar) software.

**Statistical analysis**

All statistical analyses were conducted in R (version 4.1.2, <https://cloud.r-project.org/>) or Prism 9.0. Group comparisons were carried with Fisher tests for categorical variables, Wilcoxon test for continuous variables with two groups, respectively. P value < 0.05 was considered statistically significant, and different levels were denoted as *, P < 0.05, **, P < 0.01, ***, P < 0.001 and ****, P < 0.0001, respectively. Adjusted P-value for the multiple hypothesis testing was performed by False discovery rate (FDR) method [6].

**Supplementary Figures**

**
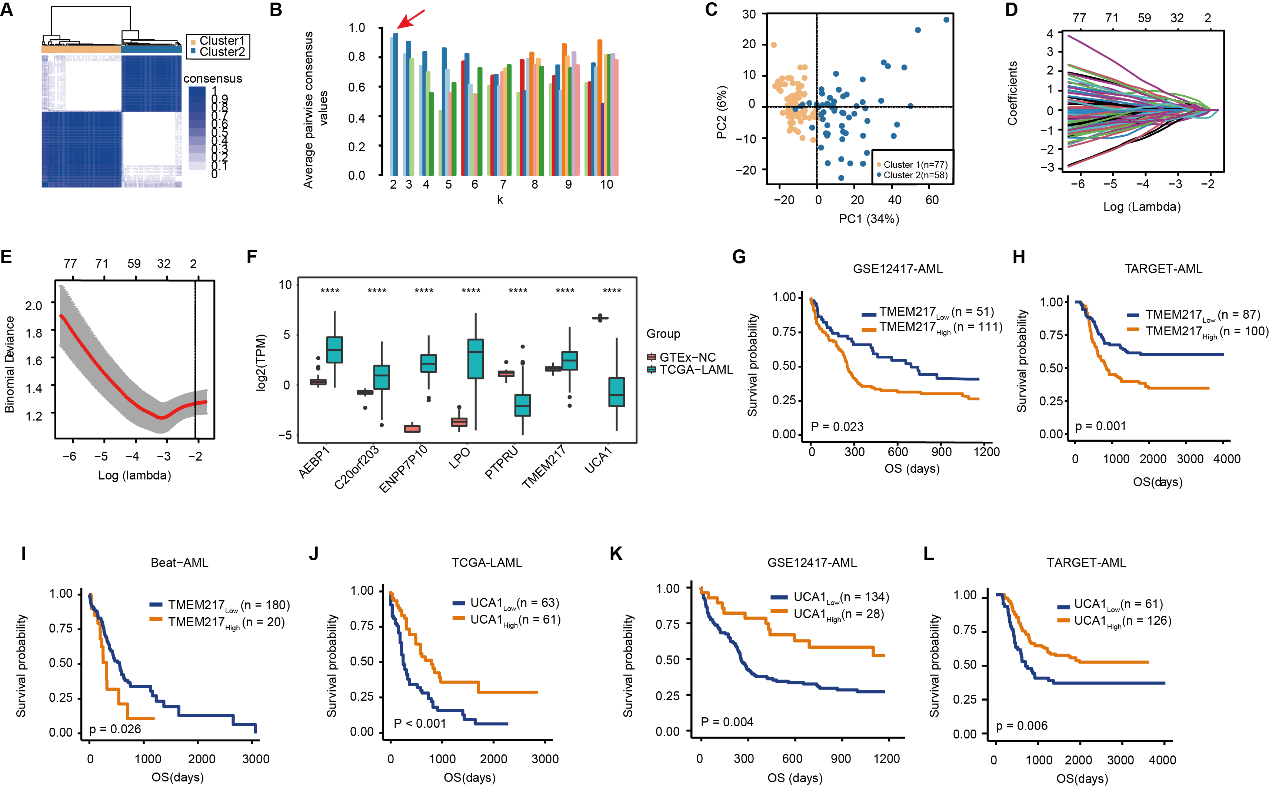
**

**Fig. S1** Feature selection identifies *TMEM217* and *UCA1* as a biomarker associated with AML patients’ prognosis. (A) The circular manhattan (CM) plot in consensus clustering analysis showed the most stable clustering when k = 2, n (Cluster 1) =77, and n (Cluster 2) = 58. (B) The cluster-consensus plot showed the average pairwise consensus values of samples at each k. The cluster-consensus plot had the highest consensus values when k = 2. The red arrow represents the k selected for analysis. (C) PCA score plots of Cluster 1 and Cluster 2 based on unsupervised consensus cluster analysis. (D) Coefficients distribution with the corresponding log(lambda) value of 10-fold cross-validation for Lasso-penalized Cox regression analysis based on 464 PRGs. PRGs, prognosis-related genes. (E) Binomial deviance distribution with the corresponding log(lambda) value of 10-fold cross-validation for Lasso-penalized Cox regression analysis based on 464 prognosis-related genes. The dotted line shows the LASSO identifies the most parsimonious model via 1-SE criteria, suggesting seven genes as optimal predictive features. (F) Seven PRGs expressions are compared between AML patients from the TCGA cohort and NC samples from the GTEx cohort, respectively. NC, normal control. (G-I) Kaplan-Meier curves for patients with high and low *TMEM217* expression in the GSE12417-AML (G), TARGET-AML (H), and Beat-AML (I) validation cohorts. Patients in the high *TMEM217* expression subgroup have worse overall survival than those in the low subgroup. Log-rank test shows an overall P value. n, number. (J-L) Kaplan-Meier curve showed among all AML patients with OS data in TCGA-LMAL (J), GSE12417-AML (K), and TARGET-AML (K) cohorts, patients in the low *UCA1* expression subgroup had worse prognostic survival than those in the high group. GSE12317-AML and TARGET-AML are set as the validation cohort. n, number.


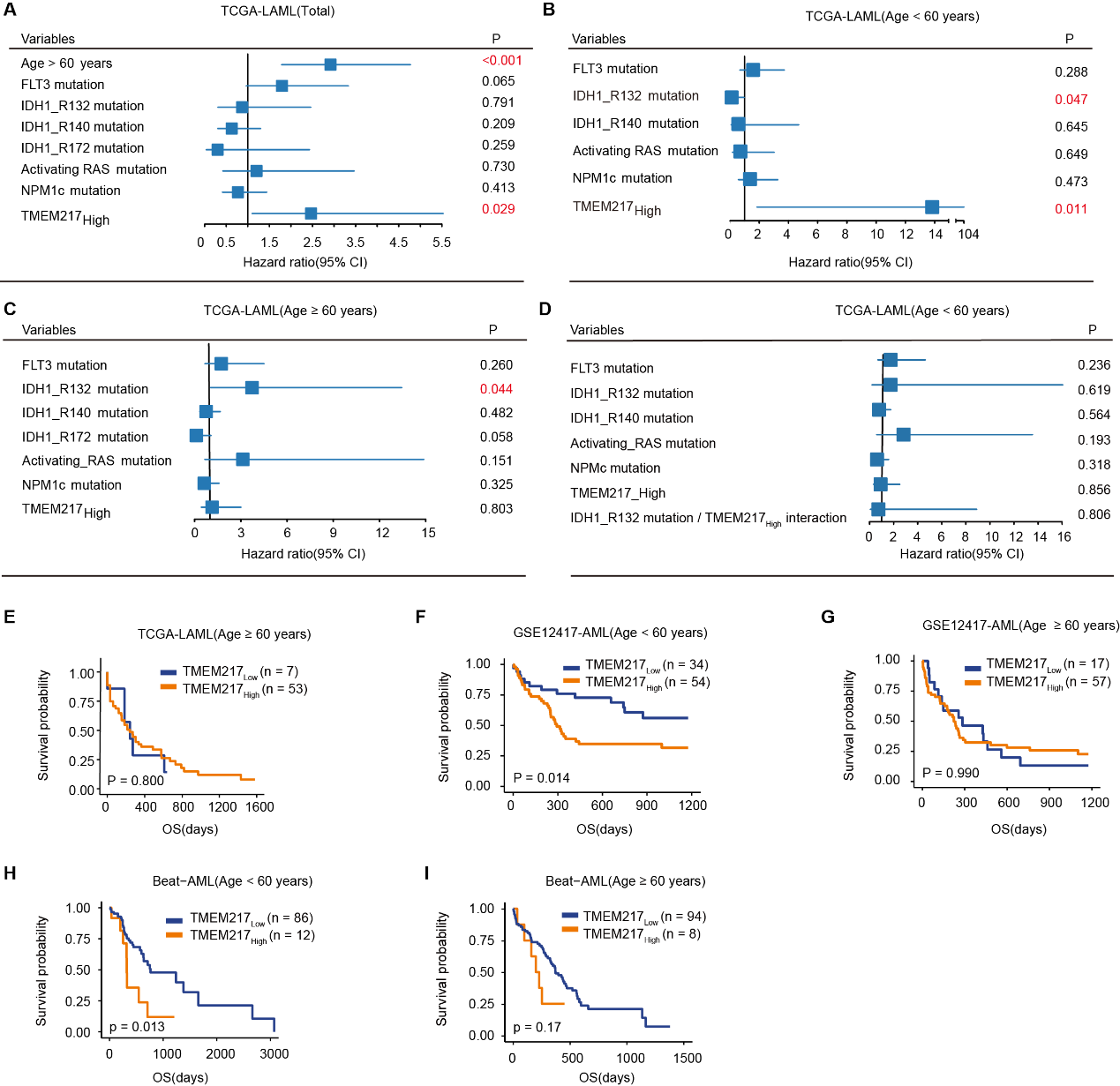


**Fig. S2** *TMEM217* expression can independently predict the prognosis of AML patients aged under 60 years. (A-D) Multivariable Cox analysis of TMEM217 and clinical characteristics regrade by age in TCGA-LAML cohort. (A) Forest plot of *TMEM217* and clinical characteristics through multivariable Cox analysis. (B) Forest plot of *TMEM217* and clinical characteristics through multivariable Cox analysis in AML patients who are younger than 60 years of age. (C) Forest plot of *TMEM217* and clinical characteristics through multivariable Cox analysis in AML patients who are older than 60 years of age. (D) Forest plot of multivariable Cox analysis to examine the interaction effects between IDH1_R132^mut^ and *TMEM217* high expression. (E-I) Kaplan-Meier curve analysis of *TMEM217* for AML patients regrade by age from TCGA-LAML, GSE12417-AML(F-G) and Beat-AML(H-I) cohorts. (E) Kaplan-Meier curve analysis of *TMEM217* for patients older than 60 years of age (age ≥ 60 years) in TCGA-LMAL cohort, which had no significant difference in OS whatever their *TMEM217* expressed (P = 0.800). (F-I) Validation of Kaplan-Meier curve analysis of *TMEM217* for AML patients regrade by age in GSE12417-AML(F-G) and Beat-AML(H-I) cohorts, the prognostic survival of younger AML patients in the *TMEM217* high expression subgroup was shorter (F, H), and older patients had similar prognostic survival probability in both groups (G, I). The TARGET-AML cohort data did not include any patients over 60 years of age.


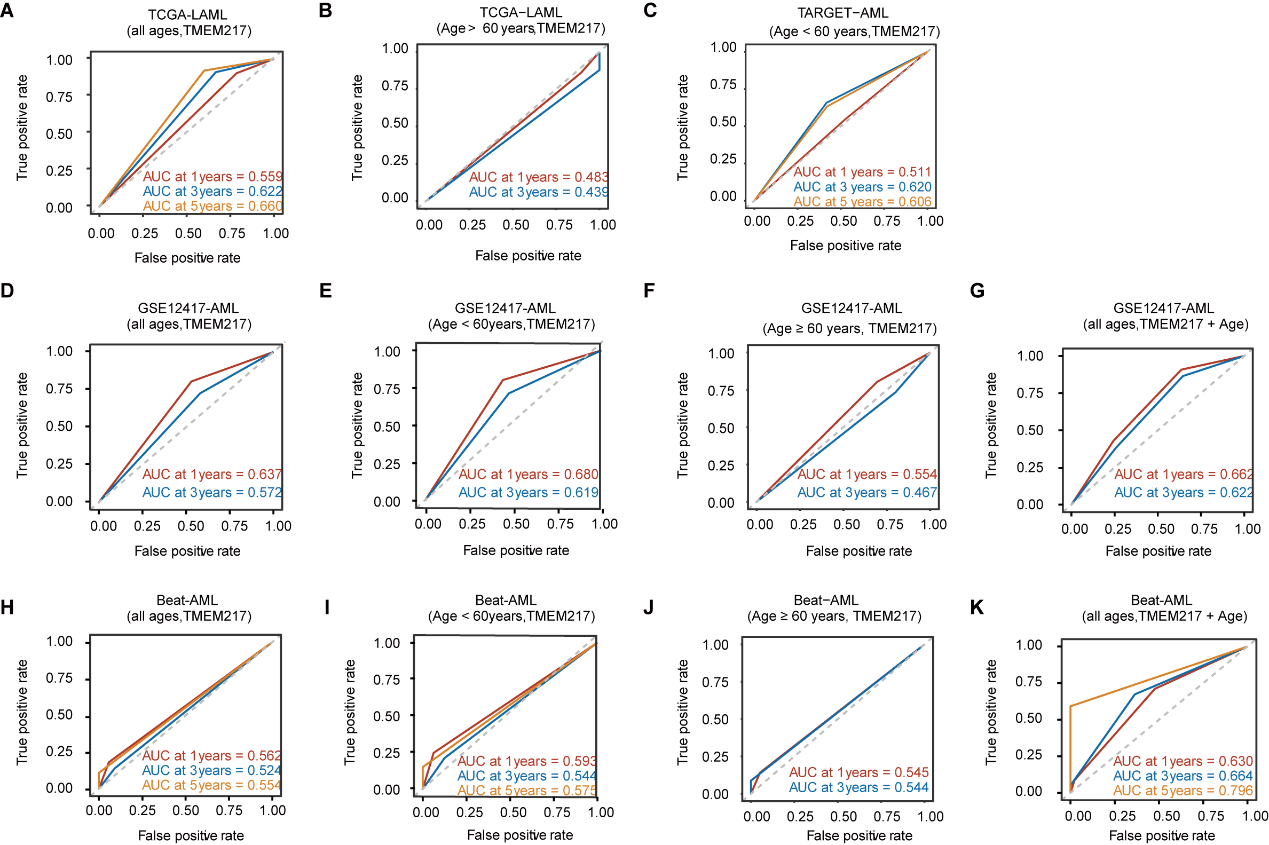


**Fig. S3** A combination of *TMEM217* expression and age can increase the accuracy of prognosis prediction by multiple Time-dependent ROC models comparing. Four models are established by the combination of three age groups (all ages, age < 60 years, age ≥ 60 years) and two predictors (age and *TMEM217*). (A-B) Time-dependent ROC curve analysis based on the expression of *TMEM217* in all ages and age ≥ 60 years groups from TCGA-LAML cohort, respectively. (C) Time-dependent ROC curve analysis was obtained using the prognostic model constructed based on *TMEM217* expression in the TARGET-AML cohort (The TARGET-AML cohort data did not include any patients over 60 years of age). (D-K) The corresponding results of TCGA-LAML were validated in the GSE12417-AML and Beat-AML cohorts, respectively.


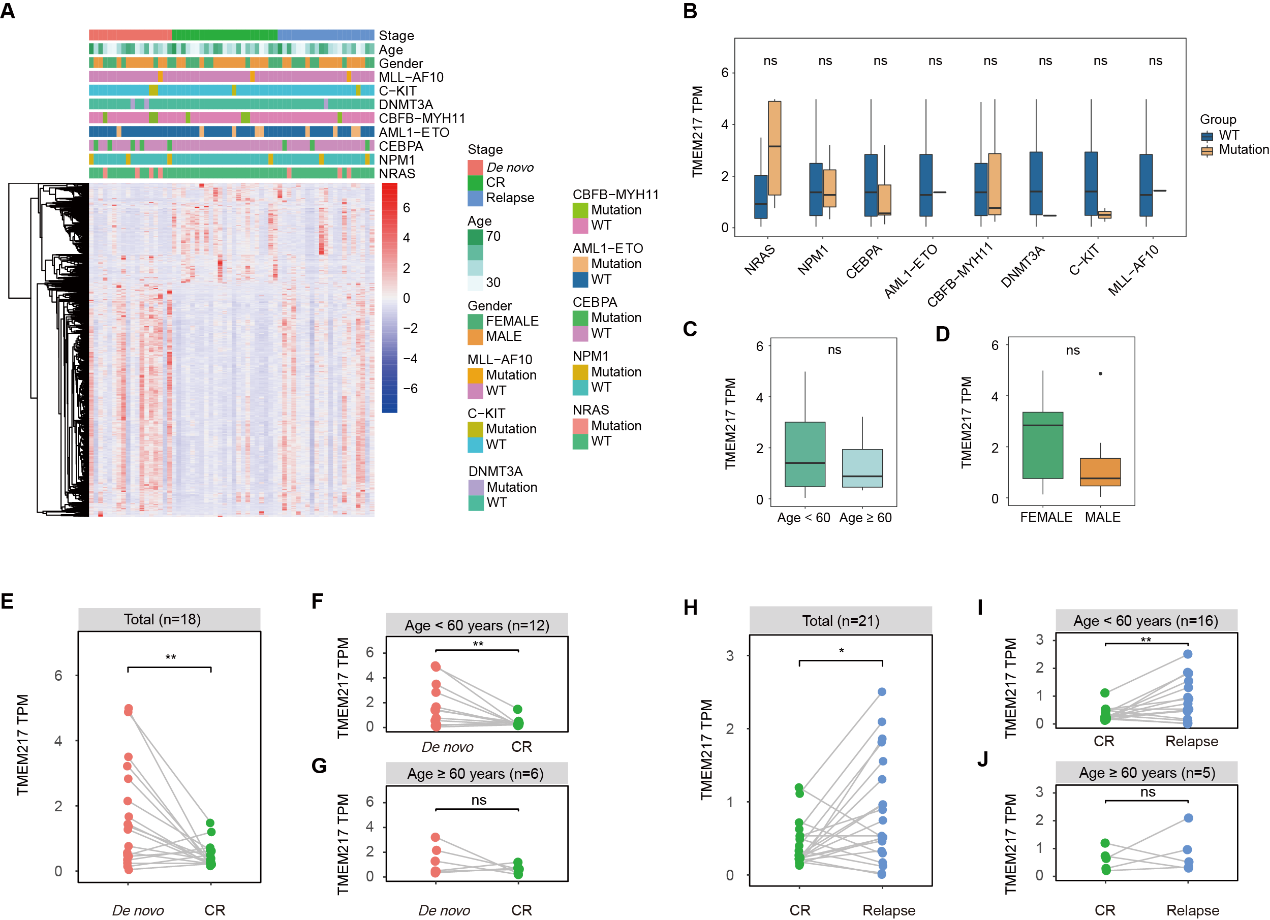


**Fig. S4** Dynamic change of *TMEM217* expression in different clinical stages of RJ-AML patients. (A) Heatmap of association between gene expression of three stages and their clinical characteristics. (B) Expression profile of *TMEM217* based on genetic mutations. (C) Expression profile of *TMEM217* based on two age subgroups. (D) Expression profile of *TMEM217* based on gender. (E-G) Pairwise comparisons of *TMEM217* expression in eighteen AML patients (E), twelve AML patients younger than 60 years of age (F), and six patients older than 60 years of age (G) who achieved CR from de novo. (H-J) Pairwise comparisons of *TMEM217* expression in twenty-one AML patients(H), sixteen AML patients younger than 60 years of age (I), and five patients older than 60 years of age (J) who achieved relapse from the CR. CR, complete remission.


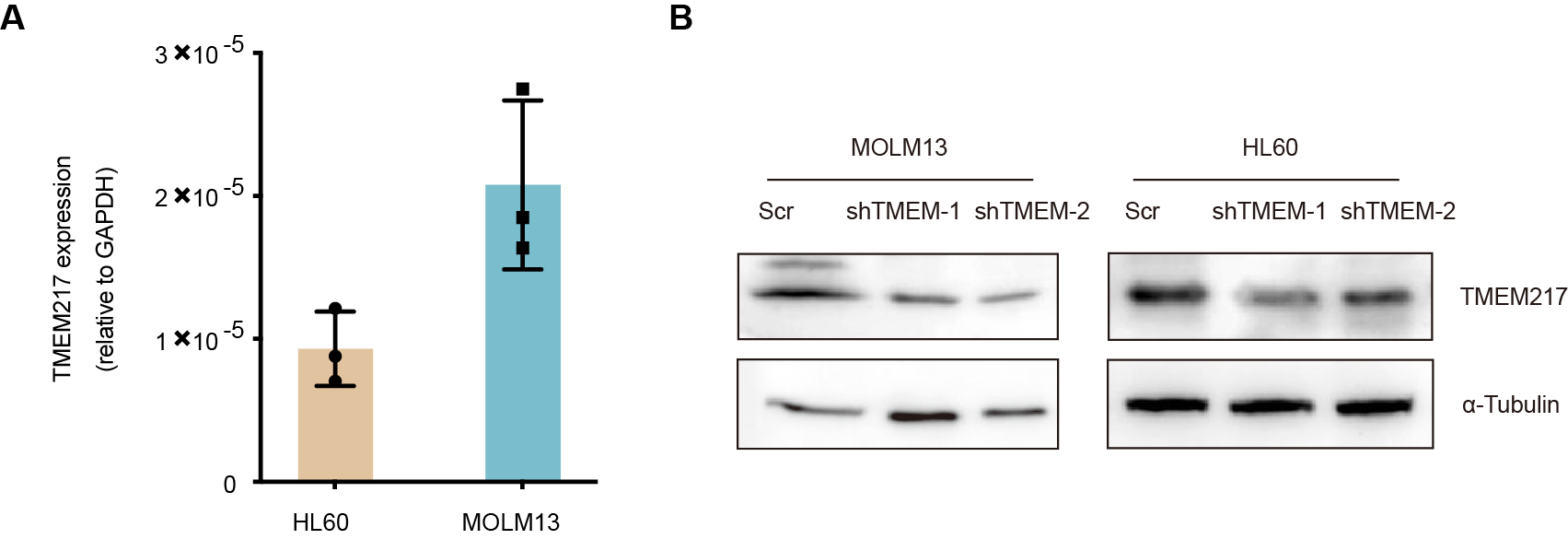


**Fig. S5** The expression and knockdown efficiency of *TMEM217* in two AML cells. (A) The mRNA expression of *TMEM217* in MOLM13 and HL60. (B) The efficiency of *TMEM217* knockdown was analyzed by Western blot. Scrambled shRNA (Scr) was used as the blank control.

**Supplementary Table**

**Table S1.** The detailed clinical characteristics of Cluster 1 and Cluster 2.

| Variables | Cluster1(n=77) | Cluster2(58) | P |
| --- | --- | --- | --- |
| Age, median (range) | 51(21,81) | 63.5(31,88) | < 0.001 |
| Cytogenetics risk category, n (%) |  | |  |
| Favorable | 10(13.33%) | 7(12.07%) | 0.322 |
| Intermediate | 42(56.00%) | 39(67.24%) |  |
| Adverse | 23(30.67%) | 12(20.69%) |  |
| FAB, n (%) |  | |  |
| M0 | 12(15.58%) | 3(5.17%) | < 0.001 |
| M1 | 22(28.57%) | 13(22.41%) |  |
| M2 | 31(40.26%) | 7(12.07%) |  |
| M4 | 7(9.09%) | 22(37.93%) |  |
| M5 | 3(3.90%) | 12(20.69%) |  |
| M6 | 1(1.30%) | 1(1.72%) |  |
| M7 | 1(1.30%) | 0(0.00%) |  |
| Vital status, n (%) |  | |  |
| Alive | 29(37.66%) | 14(24.14%) | 0.138 |
| Dead | 48(62.34%) | 44(75.86%) |  |
| Gender, n (%) |  | |  |
| FEMALE | 37(48.05%) | 23(39.66%) | 0.426 |
| MALE | 40(51.95%) | 35(60.34%) |  |
| FLT3, n (%) |  | |  |
| Negative | 56(72.73%) | 42(72.41%) | 1 |
| Positive | 21(27.27%) | 16(27.59%) |  |
| IDH1 R132, n (%) |  | |  |
| Negative | 68(88.31%) | 54(93.10%) | 0.522 |
| Positive | 9(11.69%) | 4(6.90%) |  |
| IDH1 R140, n (%) |  | |  |
| Negative | 70(90.91%) | 53(91.38%) | 1 |
| Positive | 7(9.09%) | 5(8.62%) |  |
| IDH1 R172, n (%) |  | |  |
| Negative | 77(100.00%) | 56(96.55%) | 0.357 |
| Positive | 0(0.00%) | 2(3.45%) |  |
| Activating RAS, n (%) |  | |  |
| Negative | 73(94.81%) | 54(93.10%) | 0.963 |
| Positive | 4(5.19%) | 4(6.90%) |  |
| NPM1, n (%) |  | |  |
| Negative | 64(83.12%) | 38(65.52%) | 0.031 |
| Positive | 13(16.88%) | 20(34.48%) |  |

**Additional Discussion**

The currently standard “7+3” chemotherapy regimen can efficiently induce CR of AML[14]. However, the survival probabilities of AML patients differ varied, owing to multiple reasons such as differences in age and some prognostic parameters defined upon genomics. Frequently suffered from relapse, most AML patients struggled with low overall 5-years survival rates [14-16]. Moreover, the limited benefits to OS gained from targeted drugs approved by FDA confused the clinical management [17]. Thus, there is an urgent need to discover new biomarkers to help clinicians in increasing AML survival rates.

Our transcriptome analysis based on six independent AML cohorts showed that *TMEM217* is expressed at aberrantly high levels in AML patients compared to normal controls, and *TMEM217* can be an independent biomarker to predict the prognosis of AML patients younger than 60 years of age.

*TMEM217*, located on human chromosome 6p21.2, was identified as a candidate MAPK pathway protein that contributed to the development of diabetic retinopathy via regulatory effects on steroid metabolism [18, 19]. *TMEM217* expression could be elevated by DMU-212, a derivative of resveratrol [20]. Some critical modulators of cell apoptosis and senescence such as SPT6, SIRT3, HRAS^G12V^, and FOXO3 are known to function as upstream regulators of TMEM217 [21-24]. It bears mention that according to the Human Protein Atlas, the localization of TMEM217 in HeLa (cervical carcinoma), BJ (foreskin), U-2 OS (osteosarcoma), and HUVEC/TERT2 (endothelial cell) is concentrated in the nucleus, while in HEL (a cell line derived from erythroleukemia), it is localized to the plasma membrane. These results together suggest potential differential function and/or regulatory mechanism(s) for *TMEM217* in AML compared with other carcinomas and its (as-yet-unknown) normal biological function(s).

Results from the longitudinal transcriptomics study conducted by our Ruijin center demonstrated that high *TMEM217* expression is associated with *de novo* and relapse, while low *TMEM217* expression is associated with CR for AML patients under the 60 years of age. The *TMEM217* expression level was different in *de novo* and relapse might suggest a less tumor burden in relapse compared to *de novo*. However, the sample sizes prevent our attempt from further investigation of the relationship between *TMEM217* and disease stages. We also found that knockdown of *TMEM217* decreased the proliferation and increased apoptosis of AML cells, inferring the impact of *TMEM217* in AML progression.

The current work still has some limitations to be acknowledged. Firstly, the function and molecular mechanisms of *TMEM217* affect hematopoietic malignancies should be further clarified. Additionally, although this work was conducted in six independent cohorts, the amounts of enrolled cases might lead to an analysis bias, calling for a larger validation dataset.

Our study identified *TMEM217* as a novel prognosis biomarker of AML, particularly for patients under 60 years of age. *TMEM217* combined with age had better prediction accuracy of prognosis in AML. *TMEM217* expression also associated with disease stages in AML. Finally, the impact of *TMEM217* on proliferation and apoptosis of AML cells suggested that *TMEM217* played a role in AML progression and showed potential as an AML biomarker and therapeutic target in the future.

**Acknowledgments**

We acknowledge TCGA, GTEx and GEO database for providing their platforms and contributors for uploading their meaningful datasets. We thank all the participants who contributed to our work.

**References**

1. Network N C C. NCCN Clinical Practice Guidelines in Oncology. 2022.

2. Goldman M J, Craft B, Hastie M, et al. Visualizing and interpreting cancer genomics data via the Xena platform. Nat Biotechnol 2020; 38(6):675-678.

3. Metzeler K H, Hummel M, Bloomfield C D, et al. An 86-probe-set gene-expression signature predicts survival in cytogenetically normal acute myeloid leukemia. Blood 2008; 112(10):4193-4201.

4. Tyner J W, Tognon C E, Bottomly D, et al. Functional genomic landscape of acute myeloid leukaemia. Nature 2018; 562(7728):526-531.

5. Wilkerson M D ,Hayes D N. ConsensusClusterPlus: a class discovery tool with confidence assessments and item tracking. Bioinformatics (Oxford, England) 2010; 26(12):1572-1573.

6. Benjamini Y ,Y. H. Controlling the false discovery rate: a practical and powerful approach to multiple testing. J Royal Stat Soc Series B 1995; 57:289-300.

7. Friedman J, Hastie T, Tibshirani R. Regularization Paths for Generalized Linear Models via Coordinate Descent. Journal of statistical software 2010; 33(1):1-22.

8. Heagerty P J, Lumley T, Pepe M S. Time-dependent ROC curves for censored survival data and a diagnostic marker. Biometrics 2000; 56(2):337-344.

9. Bolger A M, Lohse M, Usadel B. Trimmomatic: a flexible trimmer for Illumina sequence data. Bioinformatics 2014; 30(15):2114-2120.

10. Alexander Dobin, Carrie A. Davis, Felix Schlesinger, et al. STAR: ultrafast universal RNA-seq aligner. Bioinformatics 2013; 29(1):15-21.

11. Anders S, Pyl P T, Huber W. HTSeq--a Python framework to work with high-throughput sequencing data. Bioinformatics 2015; 31(2):166-169.

12. Xia Z, Zhang X, Liu P, et al. GNA13 regulates BCL2 expression and the sensitivity of GCB-DLBCL cells to BCL2 inhibitors in a palmitoylation-dependent manner. Cell Death & Disease 2021; 12(1).

13. Moffat J, Grueneberg D A, Yang X, et al. A lentiviral RNAi library for human and mouse genes applied to an arrayed viral high-content screen. Cell 2006; 124(6):1283-1298.

14. Hartmut Döhner, Andrew H. Wei, Frederick R. Appelbaum. Diagnosis and Management of AML in Adults: 2022 ELN Recommendations from an International Expert Panel. Blood 2022; 140(12):1345-1377.

15. Dohner H, Wei A H, Lowenberg B. Towards precision medicine for AML. Nat Rev Clin Oncol 2021; 18(9):577-590.

16. Khoury J D, Solary E, Abla O, et al. The 5th edition of the World Health Organization Classification of Haematolymphoid Tumours: Myeloid and Histiocytic/Dendritic Neoplasms. Leukemia 2022; 36(7):1703-1719.

17. Hilal T. Progress in acute myeloid leukaemia: small molecular inhibitors with small benefits. Ecancermedicalscience 2020; 14:1015.

18. Lin H J, Huang Y C, Lin J M, et al. Association of genes on chromosome 6, GRIK2 , TMEM217 and TMEM63B (linked to MRPL14) with diabetic retinopathy. Ophthalmologica 2013; 229(1):54-60.

19. Ung C, Sanchez A V, Shen L, et al. Whole exome sequencing identification of novel candidate genes in patients with proliferative diabetic retinopathy. Vision Res 2017; 139:168-176.

20. Miao Y, Cui L, Chen Z, et al. Gene expression profiling of DMU-212-induced apoptosis and anti-angiogenesis in vascular endothelial cells. Pharm Biol 2016; 54(4):660-666.

21. Nojima T, Tellier M, Foxwell J, et al. Deregulated Expression of Mammalian lncRNA through Loss of SPT6 Induces R-Loop Formation, Replication Stress, and Cellular Senescence. Molecular cell 2018; 72(6).

22. Diao Z, Ji Q, Wu Z, et al. SIRT3 consolidates heterochromatin and counteracts senescence. Nucleic acids research 2021; 49(8):4203-4219.

23. Casella G, Munk R, Kim K M, et al. Transcriptome signature of cellular senescence. Nucleic acids research 2019; 47(14):7294-7305.

24. Yan P, Li Q, Wang L, et al. FOXO3-Engineered Human ESC-Derived Vascular Cells Promote Vascular Protection and Regeneration. Cell stem cell 2019; 24(3).
